# Current Trends in Fetal Cardiology: Results from an International Benchmarking Survey of Fetal Cardiac Programs Through the Fetal Heart Society Research Collaborative

**DOI:** 10.1007/s00246-025-03895-5

**Published:** 2025-05-29

**Authors:** Sheetal Patel, Angela McBrien, Erik Michelfelder, Ann Kavanaugh-McHugh, Stefani Samples, Christina Laternser, Lisa Hornberger, Anita Moon-Grady, Shubhika Srivastava, Jacqueline Shuplock

**Affiliations:** 1 Division of Pediatric Cardiology, Ann & Robert H Lurie Children’s Hospital of Chicago, Northwestern University Feinberg School of Medicine, 225 E Chicago Ave, Box 25, Chicago, IL 60611 USA; 2 Fetal and Neonatal Cardiology Program, Division of Pediatric Cardiology, Department of Pediatrics, University of Alberta, WMC 4 C2, 8440 112 St, Edmonton, Alberta T6G 2B7 Canada; 3 Fetal Cardiology Program, Children’s Healthcare of Atlanta Cardiology, Emory University School of Medicine, Atlanta, GA 30341 USA; 4 Division of Cardiology, Monroe Carell Jr Children’s Hospital at Vanderbilt, Nashville, TN 37232 USA; 5 Division of Cardiology, University of California San Francisco, San Francisco, CA USA; 6 Division of Cardiology, Nemours Children’s Health, Wilmington, DE 19803 USA; 7 Division of Pediatric Cardiology, AdventHealth for Children, Orlando, FL 32804 USA

**Keywords:** Fetal cardiac program, Benchmarking, Quality improvement

## Abstract

Fetal cardiology has grown into a robust pediatric cardiology subspecialty in the last two decades, with many congenital heart centers having a dedicated fetal cardiac program. Despite the subspeciality’s clear maturation, there are no multicenter data to describe volume, practice patterns, program structures, resources, or trends. The Fetal Heart Society sought to address this deficiency by conducting an international survey of fetal cardiac programs. A survey was distributed internationally to fetal cardiac programs. One response per institution or clinical practice was collected. Respondents were asked to provide data from the 2022 calendar year. Ninety-five programs responded, with the majority representing the United States of America (n = 75, 79%) or Canada (n = 11, 12%). Most responding programs (88%) had an academic or university affiliation, and 69% were from independent children's hospitals. The median number of fetal echocardiographic studies performed annually per program was 1,000 (IQR 580–2,100). There was a median of 5 (IQR 3–7) fetal cardiologists and 5 (IQR 3–7) sonographers per program. Each fetal cardiologist interpreted an estimated median of 6 (IQR 5–8) fetal studies in a full day shift. The most common duration allocated for each fetal echocardiographic study was 45–59 min for the initial study (51%) and 45–59 min for the follow-up study (42%). 79% of programs had a fetal cardiac nurse coordinator. An independent fetal database was maintained at 78% of programs. Less than half of programs (46/95) had a formal quality improvement (QI) initiative, with only 22 programs participating in national-level QI metrics. The most frequently reported barrier to having a fetal cardiac QI program was a lack of human resources (60%), followed by a lack of institutional support/incentive (41%). Programs were more likely to have a formal fetal cardiology QI program if they had a fetal cardiac coordinator (p = 0.0029) if they had a formal fetal database (p = 0.003), or if they were a larger volume program (p = 0.026). Certain subspecialties were available at most programs, including neonatology (93%), maternal–fetal medicine (88%), genetic counseling (88%), and social work (79%). However, psychology (38%) and psychiatry (16%) services to address parental mental health issues were not as commonly available. These survey data provide a novel and comprehensive view of fetal cardiology programs with information useful for internal benchmarking, quality improvement initiatives, resource allocation, and identifying unmet needs.

## Introduction

In the developed world, most cases of critical congenital heart disease (CHD) are now detected prenatally, revolutionizing pathways of care for these patients. Fetal cardiology has grown into a notable pediatric cardiology subspecialty focusing on the prenatal detection and perinatal management of CHD. The duties of a fetal cardiologist include performing and interpreting fetal echocardiographic studies, providing prenatal counseling, guiding prenatal and perinatal management, and planning the personalized CHD interventions and resources needed after birth. Most congenital heart centers, therefore, either have a dedicated fetal cardiac program with specialized fetal cardiology teams and resources or are in the process of developing a fetal cardiac program that focuses on prenatal detection of CHD by fetal echocardiography and subsequent perinatal management of CHD lesions. Previously, other pediatric cardiac societies such as the Society of Cardiovascular Magnetic Resonance (SCMR) [1] and the American Society of Echocardiography (ASE) [2–5] have periodically surveyed their respective specialties to gather benchmarking data related to practice components, productivity, and resource allocation. However, there is a paucity of such multicenter data describing fetal cardiology practice patterns and trends. This lack of data limits the ability of a program to review its practices, evaluate its administration, and identify unmet needs compared with other centers.

The Fetal Heart Society (FHS) is a nonprofit organization formed in 2014 with the mission to advance fetal cardiovascular care and science through collaborative research, education, and mentorship. With the support of FHS, an international survey of fetal cardiologists and fetal cardiac programs was created. The survey aimed to collect information on fetal cardiology practices to help understand current trends in fetal cardiology program structures and workflows. The survey data will serve as a resource for program development, gap analysis, quality improvement initiatives, and community activities, with a plan for future follow-up surveys to track emerging trends.

## Methods

### Survey Design

The study authors compiled and modified the survey based on input from the FHS Program Leaders Committee members. The survey collected information on the following:

1. Program characteristics: location, hospital type (dedicated/independent children’s hospital, integrated with adult/obstetric services, or outpatient practice), academic/university affiliation, accreditation status, availability of maternal delivery services, and cardiac surgical volume.
2. Staffing: number of fetal cardiologists, number of fetal cardiac sonographers, sonographer specialty type (pediatric echocardiographer versus obstetric sonographer), number of fetal cardiac coordinators and their obligations, number of pediatric cardiology fellows and involvement of categorical pediatric cardiology fellows in fetal cardiology.
3. Fetal cardiology patient volume: annual number of fetal echocardiographic studies performed and number of locations offering fetal cardiology services.
4. System or institutional practices: time allotted per fetal study, physician presence during studies, use of telehealth fetal services, echocardiography machine number and use, image analysis and structured reporting platform, fetal echocardiographic techniques used for clinical or research purposes (e.g., 3-dimensional, early gestational fetal echo, myocardial strain, others), use of acute maternal hyperoxia testing, use of fetal magnetic resonance imaging (MRI), availability of fetal cardiac and non-cardiac interventions, availability of additional fetal multidisciplinary services (e.g., genetics, neonatology, social work, others), maintenance of fetal database, participation in quality improvement initiatives, use of level of care (LOC) and high-risk delivery protocols, and interest in participating in future surveys.

### Survey Distribution

After approval by the Northwestern University Feinberg School of Medicine Institutional Review Board, the survey was distributed to FHS members globally via REDCap (hosted by Ann & Robert H. Lurie Children's Hospital of Chicago). To identify programs not included in FHS, internet searches and/or direct contact with fetal cardiology programs were performed, and the survey was distributed to those programs. One response per program was collected. Respondents were asked to provide data from the 2022 calendar year.

### Statistical Methods

All responses to this survey were de-identified, and only aggregate data were described as survey results in the form of descriptive statistics. Continuous variables were noted as medians and interquartile range. Bivariate analysis was performed using correlation statistics. A p-value of < 0.05 was considered statistically significant.

## Results

### Program Characteristics

A total of 95 fetal cardiology programs responded to the survey. Programs submitted data from the 2022 calendar year. The survey respondents were most often fetal cardiologists (n = 89, 94%), specifically the fetal cardiology program director (n = 59, 62%).

#### Location

The countries represented included the United States of America (n = 75, 79%), Canada (n = 11, 12%), and one program each from Austria, Germany, Hong Kong, Italy, Japan, Mexico, Poland, the United Kingdom, and Vietnam. The individual respondents and the institutions they represent are listed in the acknowledgment section.

#### Hospital Setting

The majority of responding fetal cardiac programs (n = 66, 69%) classified their hospital or practice setting with pediatric cardiology services as a freestanding/independent children’s hospital. A combined setting with pediatric and maternal services within one institution or administration occurred in 25 programs (26%). Four programs (4%) were classified as outpatient practices. Most programs (84/95, 88%) were affiliated with an academic institution or university.

#### Accreditation Status

For programs in the U.S., 67 (89%) were accredited by the Intersocietal Accreditation Commission and two (3%) by the American Institute of Ultrasound in Medicine. Four programs (5%) did not have accreditation, and two (3%) were unsure of their accreditation status.

#### Delivery Services (n = 88)

Out of the 88 programs that provided childbirth and delivery services or resource information, 60 (68%) had maternal delivery services available in the same hospital where pediatric cardiology services were provided; however, the level of obstetric (OB) care coverage was variable. 15 of these 60 programs (25%) allowed only planned deliveries of fetuses with complex CHD via cesarean delivery. The remaining 45 programs (75%) had a delivery unit within the same institution as the pediatric cardiology center with continuous OB coverage; they allowed either vaginal or cesarean deliveries based on fetal or OB indications.

Maternal delivery services were in a separate location in 28 out of 88 programs (32%), mainly the freestanding/independent children's hospitals without integrated OB services. At these centers, neonates with CHD would be delivered at the maternal hospital and then transferred to the children's hospital for pediatric cardiac care. The distance between the children's hospital and the delivery unit was variable, with 64% (n = 18/28) connected via a walking bridge or a tunnel, allowing neonate transport without requiring an ambulance. The remaining ten pediatric cardiology programs were located more than one mile from the maternal delivery unit and thus required an ambulance to transport the neonate.

#### Surgical Volume

Most centers participating in this survey (70/80, 88%) offered congenital heart surgery. The annual case volume in these 70 congenital heart surgery programs for 2022 was as follows: 10(14%) of centers performed ≤ 150 cases, 23 (33%) performed 151–300 cases, 21 (30%) performed 301–500 cases, and 16 programs (23%) performed ≥ 501 cases. Sixty five (65/70; 93%) of these programs performed all types of congenital heart surgery without referring cases elsewhere. Neonatal extracorporeal membrane oxygenation was offered at 57 (81%) of centers, ventricular assist device program at 44 (63%), and cardiac transplantation at 43 (61%) surgical programs.

### Staffing

#### Number of Fetal Cardiologists

The median number of fetal cardiologists per program was 5 (IQR 3–7), ranging from one to 22 cardiologists per program.

#### Number of Fetal Cardiac Sonographers and Specialty Type (n = 80)

The median number of fetal cardiac sonographers per program was 5 (IQR 3–7), ranging from no fetal cardiac sonographers to 22 per program.

Most responding programs (64/80, 80%) reported that sonographers perform all fetal echocardiographic studies. Ten percent (8/80) of programs had no sonographer support, with fetal cardiologists performing all fetal echocardiographic studies. The remaining 10 percent (8/80) had partial sonographer support, with some studies performed by the sonographer and some by the fetal cardiologist.

Sonographers performing fetal echocardiography had variable training backgrounds. Pediatric cardiac sonographers trained in fetal echocardiography were present in 66 out of 80 responding programs (83%), maternal–fetal medicine (MFM) sonographers also capable of performing fetal echocardiograms were present in 22 out of 80 responding programs (28%) and dedicated fetal cardiac sonographers who only performed fetal echocardiograms were present in 11 out of 80 responding programs (14%). Notably, many programs had combinations of pediatric and MFM-trained sonographers trained in fetal echocardiography, as reflected in the above percentages.

#### Fetal Cardiac Coordinators (n = 80)

Most (n = 63/80, 79%) responding programs reported having one or more fetal cardiac coordinators. Most of these programs (50/63, 79%) had registered nurses (RNs) in the fetal cardiac coordinator role. The remaining 13 programs had either advanced practice registered nurses (APRNs) or physician assistants (PAs) in the role. Programs provided a median of 1 (IQR 0.7–1.4) full-time equivalent (FTE) for fetal cardiac coordinator support, ranging from 0 to 4.8 FTE. In some programs, the total approved coordinator FTE was shared among multiple part-time coordinators or coordinators with shared roles in two or more subspecialties. The median number of RN fetal cardiac coordinators per program was 1 (IQR 1–2), ranging from no RN coordinators to 8 per program. APRN or PA fetal cardiac coordinators were less represented (13 programs), with a median per program of 0 (IQR 0–0.5), ranging from no APRN/PA coordinators in a program to 2 per program. Additional MFM fetal coordinator support was available in approximately half the programs, with a median of 1 MFM coordinator per program (IQR 0–1; range 0–6).

Fetal cardiac coordinator responsibilities, as reported by the 63 programs (listed in order of frequency), included care coordination (n = 58, 92%), assisting with communication regarding delivery planning (n = 55, 87%), patient scheduling (n = 42, 67%), quality improvement database support (n = 39, 62%), research database support (n = 35, 56%), providing patient counseling (n = 13, 21%), and seeing patients as an independent provider (n = 6, 10%). Other responsibilities included providing tours of the neonatal/cardiac intensive care unit, enlisting parents in support services, screening and triaging incoming referrals, scheduling multidisciplinary visits, coordinating the transfer of care, family support, and continuity of care through the first year of the child's life.

Univariate logistic correlation analysis demonstrated that programs with fetal cardiac coordinators are more likely to have formal QI processes (p = 0.0029).

#### Number of Pediatric Cardiology Fellows and Fellow Involvement (n = 80)

Two-thirds (n = 53/80, 66%) of responding programs had categorical pediatric cardiology fellows, and 16% (13/80) had advanced non-invasive imaging fellows. Out of the 53 programs with categorical pediatric cardiology fellows, all allowed each categorical fellow to learn fetal echocardiography and observe counseling based on interest. Most of these 53 programs (n = 37, 70%) reported that categorical fellows specifically interested in fetal cardiology were provided hands-on experience performing the fetal echocardiogram. The remaining 16 (30%) reported that all categorical fellows (regardless of interest) were exposed to hands-on fetal echocardiogram experience.

Fellow observations of counseling were more mixed. All categorical fellows were allowed to observe fetal counseling in 40% (21/53) of programs, but in 43% (23/53), observation of counseling was reserved specifically for those interested in fetal cardiology as a future career path. The rest (17%) of the respondents did not specify fellow observation capabilities.

### Fetal Cardiology Volume

#### Volume of Fetal Echocardiographic Studies

The median number of fetal echocardiographic studies performed annually per program was 1000 (IQR 580–2100; range 120–5097). Most studies were conducted at the main hospital site (median 634; IQR 350–1036; range 0–3200), followed by outreach sites (median 180; IQR 50–433; range 0–3005) and MFM/OB sites (median 135; IQR 0–467; range 0–2300). Higher annual fetal echo volume correlated with a higher number of fetal cardiologists (p < 0.001) and a higher number of sonographer staffing (p < 0.001).

The survey did not collect data on fetal echocardiographic study interpretation volume per cardiologist, as this survey was program-specific and not physician-specific. However, to roughly estimate the median number of fetal echocardiographic studies interpreted annually per fetal cardiologist, the number of fetal echocardiographic studies per program was divided by the number of fetal cardiologists at that specific program. Based on this calculation, the estimated median number of fetal echocardiographic studies interpreted annually per fetal cardiologist was 220 (IQR 136–310), with a median of 6 studies per day (IQR 5–8).

Similarly, the estimated median number of fetal echocardiographic studies performed annually per fetal cardiac sonographer was determined as described above. Based on this calculation, the estimated median number of fetal echocardiographic studies performed annually per fetal cardiac sonographer was 200 (IQR 140–314), with a median of 6 studies performed per day (IQR 5–8).

#### Locations Offering Fetal Cardiology Services

The number of sites or locations offering fetal echocardiograms per program ranged from 1 to 16, with a median of 3 sites per program. Locations included the main hospital, outreach, MFM/OB, and telehealth sites.

### System or Institutional Practices

#### Time Allotted per Study

**Fig. 1:**
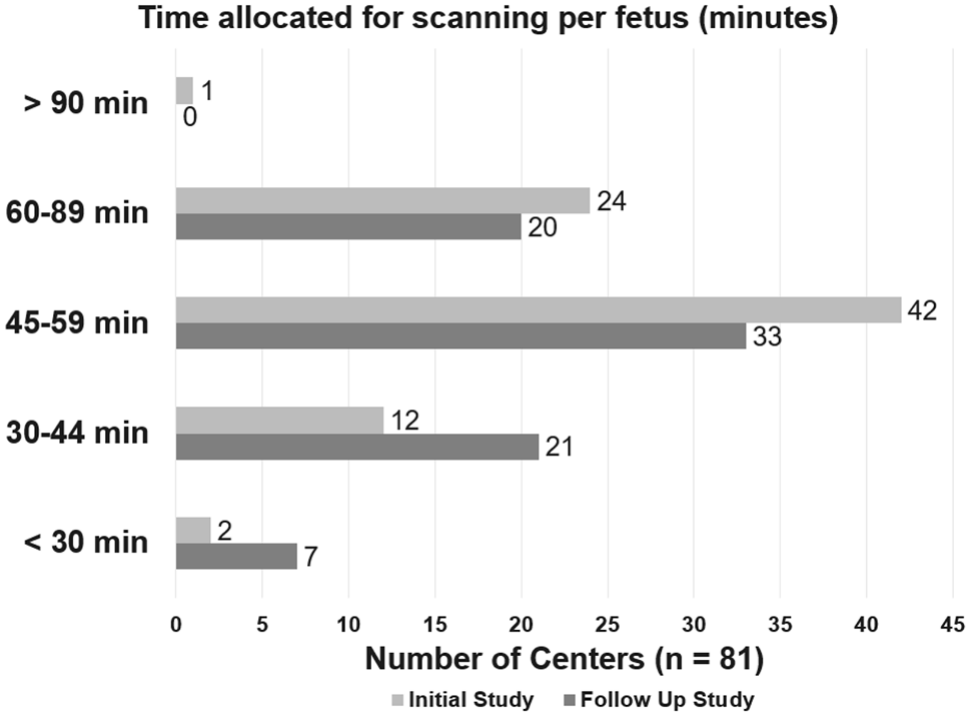
Time allocated per initial and follow-up fetal echocardiographic studies. Percentage of programs reporting the average time allocated for both an initial and follow-up fetal echocardiographic study. *Min* minutes

The time allotted for each fetal echocardiogram was variable between programs; however, most programs allocated 45–59 min for an initial echocardiogram (42/80; 51%) and 45–59 min for a follow-up echocardiogram (34/82; 42%) as shown in Fig. 1.

#### Telehealth Fetal Services (n = 95)

Just over one-third (n = 32/95, 34%) of programs offered telehealth fetal cardiology services, with the fetal cardiologist remotely interpreting the fetal echocardiograms and providing telehealth patient counseling. Of these 32 telehealth programs, 16 (50%) reported that the fetal cardiologist could review images in real time and guide the sonographer if more images were needed. On the other hand, 15 (47%) reported that the fetal cardiologist reviewed the images later, but there was no opportunity to guide the scan in real time. The remaining program utilized the fetal echocardiographic images obtained at the initial center and provided telehealth services for second opinions and virtual counseling.

#### Echocardiography Machine Number and Use (n = 95)

In addition to being used for fetal echocardiograms, many ultrasound machines are shared among pediatric cardiology divisions for pediatric transthoracic and transesophageal echocardiograms and with MFM/OB teams. Programs had a median of 1 machine reserved solely for fetal echocardiography (range 0–9), a median of 2 machines shared with pediatric cardiology (range 0–15), and a median of 1 machine shared with MFM/OB teams (range 0–6).

#### Image Analysis and Structured Reporting Platforms (n = 95)

Different image analysis and structured reporting platforms are available for fetal echocardiography. Syngo by Siemens Healthineers (40%) and Philips Intellispace Cardiovascular (27%) were the two most reported image analysis and structured reporting platforms. Other less frequently reported platforms included AS reporting system (AS Software; 4%), ViewPoint Ultrasound (GE Healthcare; 4%), Lumedx (Intelerad Medical Systems; 4%), McKesson (4%), Emeris (2%), VidiStar (VidiStar LLC; 2%), and one response each for ConnectCare (MidMichigan Health), AGFA Enterprise Imaging/Ascend (AFGA HealthCare), Epic Cupid (Surety Systems), PACS, Synapse 3D (Fujifilm Healthcare), IMPAX, PICOM, Astreia, TOMTEC, Synapse, and Digisonics (Intelerad Medical Systems).

#### Fetal Echocardiographic Modalities (n = 82)

**Fig. 2:**
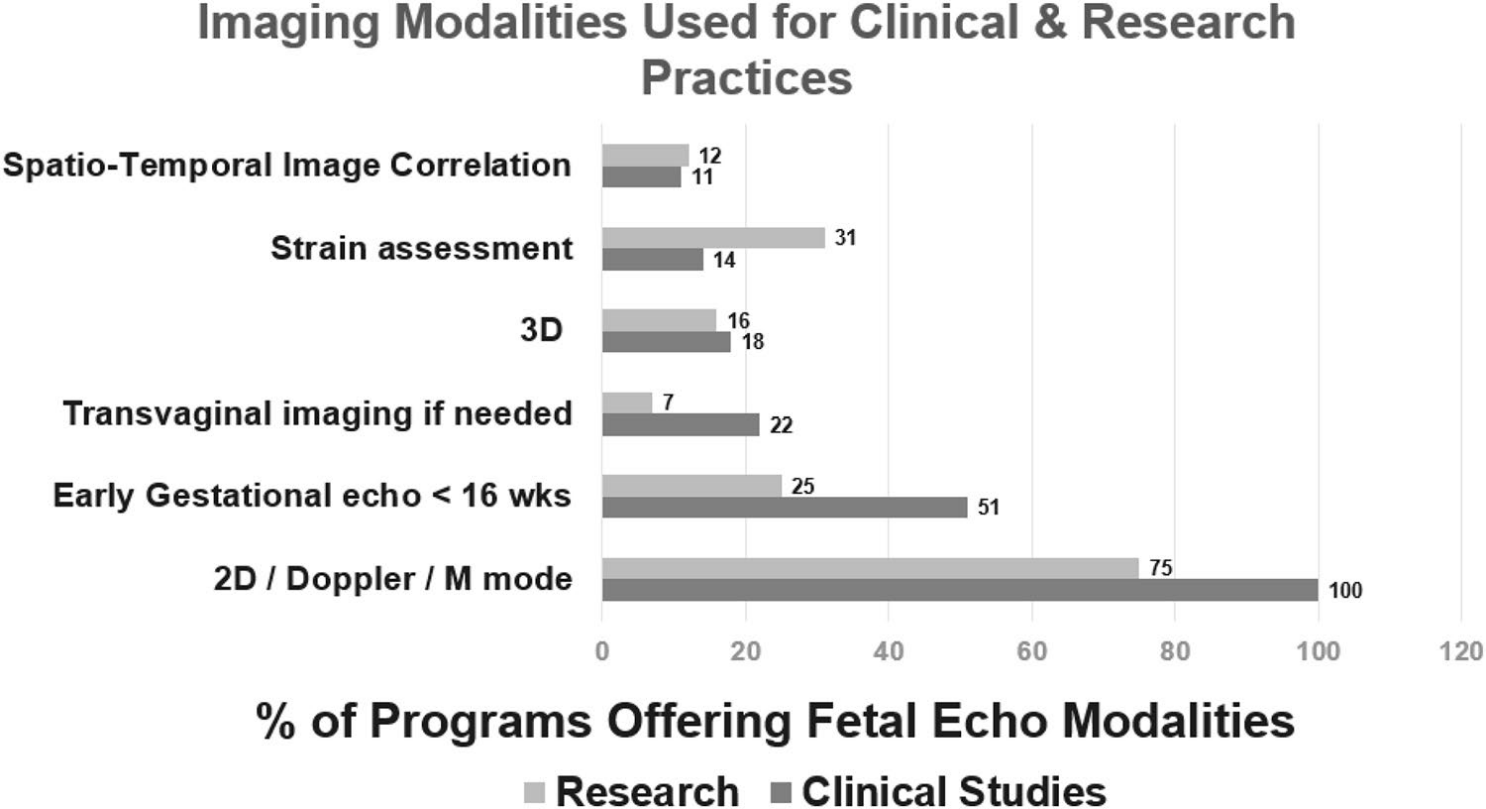
Percentages of programs using advanced imaging techniques for clinical and research practices (Number of responders = 82).Percentage of programs using advanced imaging techniques for clinical and research practices. *3D* three-dimensional, *wks* weeks, *2D* two-dimensional *M mode* motion mode

Two-dimensional (2D), Doppler and motion mode (M-mode) remain the standard of care in clinical practice. However, newer techniques are emerging as both clinical and research tools (Fig. 2). Early gestation fetal echocardiography (less than 16 weeks gestation) was offered at 42 out of 82 responding programs (51%) as a clinical tool, and 18 out of 82 (22%) used transvaginal imaging if needed. Three-dimensional (3D) imaging (n = 15/82, 18%) and fetal myocardial strain assessment (n = 12/82, 15%) were more often used as research tools.

#### Acute Maternal Hyperoxia Testing (n = 82)

Acute maternal hyperoxia (MHO) testing was offered at 29 out of 82 responding programs (35%), either for clinical use at 27 out of 82 (33%) and/or for research purposes at 17 out of 82 (21%), with overlaps of clinical and research use reflected in the percentages listed above. For programs that did not offer MHO testing, 22 were interested in starting, and 23 were either not interested or needed more resources before starting an MHO testing program at their center.

The programs using MHO reported that the hyperoxygenation protocol adds a median of 23 min (IQR 0–30 min) to the baseline fetal echocardiogram.

#### Fetal MRI (n = 83)

Fetal cardiac MRI was used for fetal cardiac evaluation in 14 out of 83 responding programs (17%), and fetal body MRI was used for extracardiac assessment in 69 out of 83 responding programs (83%).

#### Fetal Cardiac and Non-cardiac Interventions

Nine programs offered fetal cardiac interventions: seven from the USA, one from the UK, and one from Canada. Of those, eight offered fetal aortic balloon valvuloplasty, six offered fetal balloon atrial septostomy or stenting, and five offered fetal pulmonary balloon valvuloplasty. The survey did not ask about fetal cardiac procedure volumes at these centers.

Fetal non-cardiac interventions were offered at 51 programs, including the ex-utero intrapartum treatment procedure for airway obstruction at 46 programs, laser photocoagulation for twin-twin transfusion syndrome at 33 programs, fetal myelomeningocele repair at 22 programs, and the fetoscopic endoluminal tracheal occlusion procedure for congenital diaphragmatic hernia at 14 programs.

#### Fetal Multidisciplinary Services

**Fig. 3:**
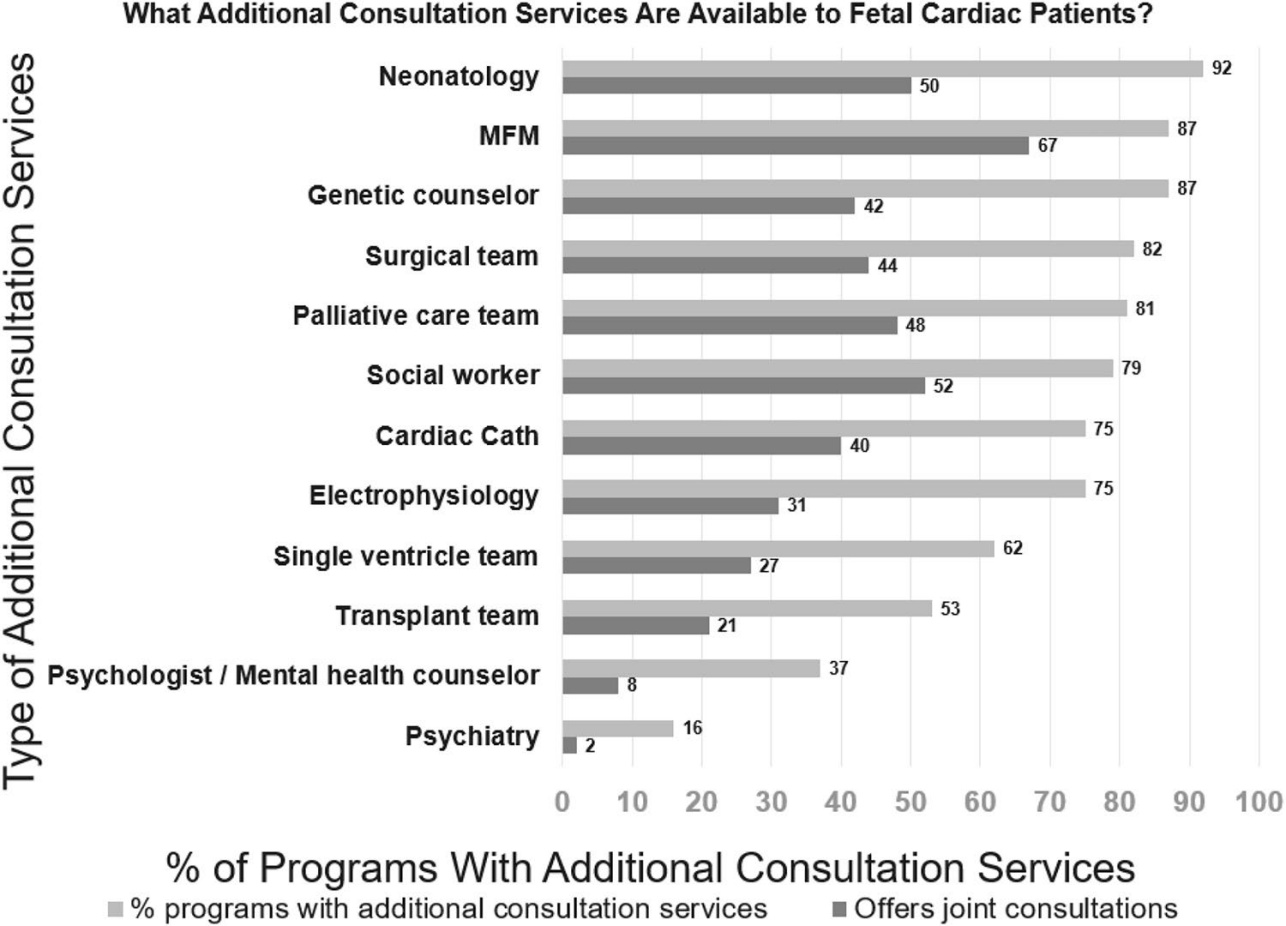
Percentage of programs with fetal multidisciplinary services available. Percentage of programs with fetal multidisciplinary services available, either as individual counseling or joint counseling with the fetal cardiology team. *MFM* maternal fetal medicine

Fetal medicine is a multidisciplinary specialty. Out of the 80 programs that provided multidisciplinary resource information (Fig. 3), the highest-represented disciplines included neonatology, MFM, genetics, cardiac surgery, palliative care, social work, electrophysiology, single ventricle care, and cardiac transplant, all of which were present at more than 50% of the 95 programs. The least represented disciplines were mental health counseling/psychology (n = 30/80, 38%) and psychiatry (n = 13/80, 16%).

Specialists may provide prenatal consultation individually or as part of a multidisciplinary consultation. Nearly two-thirds of programs (n = 58, 61%) offered joint or simultaneous counseling with fetal cardiology at the same clinic visit, which commonly included MFM, social work, neonatology, palliative care, cardiac surgery, and genetics.

#### Fetal Database (n = 64)

Most programs (64/82; 78%) maintained an independent fetal database. Of those programs, Excel (n = 37/64, 58%) and REDCap (n = 17/64, 27%) were the most used software to maintain the fetal database. Others included the Western Canadian database, Lumedx (Intelerad Medical Systems), SharePoint (Microsoft), EPIC, FileMaker (Claris International), Abacus, Microsoft Access, Tricefy (Trice Imaging), and internally-built databases.

The person/process primarily responsible for the fetal database was the fetal cardiac coordinator (48/64; 75%), fetal cardiologist (22/64; 34%), sonographer (10/64; 16%), research assistant (4/64; 6%), MFM coordinator (2/64; 3%), fourth-year imaging fellow (2/64; 3%), automatic data extraction (2/64; 3%), administrative assistant (2/64; 3%), a clinic team member other than the fetal cardiac coordinator (1/64; 2%), program manager (1/64; 2%) and/or echo coordinator (1/64; 2%). As reflected in the percentages above, some centers share the primary responsibility of maintaining such a database between different team members.

#### Quality Improvement (n = 80)

The majority of programs (89%) reported accreditation by IAC, which requires certain QI processes. However, just over half (n = 46/80, 58%) of programs responded that they had a formal QI program in place beyond the basic measures mandated by IAC. Of those 46, 22 programs participated in a national-level QI program, with 15 participating in the American College of Cardiology—Adult Congenital & Pediatric Quality Network (ACPC-QNet), 6 in the International Fetal Cardiac Intervention Registry, and 2 in the Cardiovascular Neurodevelopmental Outcome Collaborative (CNOC). For the 15 centers participating in ACPC-QNet, only eight programs submitted their data regularly, two submitted data occasionally, and five did not submit their data and only used the ACPC-QNet metrics for internal QI processes. The reported barriers to submitting data to ACPC-QNet were a lack of monetary support for participation and a lack of support for data entry.

The person primarily responsible for conducting the QI program was the fetal cardiology program director (23/46; 50%), another fetal cardiologist (14/46; 30%), a sonographer (6/46; 13%), or a fetal cardiac coordinator (1/46; 2%) and others (2/46; 4%). A QI program is often a team effort, and commonly, additional sonographers, physicians, fetal cardiac coordinators, and/or pediatric cardiology fellows assist with it.

Commonly reported barriers to establishing fetal cardiac quality assurance and quality improvement programs included lack of human resources for data keeping and data entry (48/80; 60%), lack of institutional support or incentive for participating in QI (33/80; 41%), lack of infrastructure such as information technology (IT) or database support to collect and analyze data (28/80; 35%), and/or lack of guidance or skill set (9/80; 11%). Correlation analysis showed programs are more likely to have formal QI processes if they have fetal cardiac coordinators (p = 0.0029), a formal fetal database (p = 0.003), or larger annular fetal echocardiographic volume (p = 0.0265).

#### Level of Care and Delivery Guidelines (n = 80)

More than half of the responding programs (41/80; 51%) used perinatal LOC guidelines. The remaining programs reported they did not have LOC guidelines (29/80; 36%) or were unfamiliar with them (10/80; 13%). Formal processes or algorithms for delivering fetuses with high-risk CHD were used in 71% (57/80) of programs.

## Discussion

This is the first fetal cardiology program benchmarking survey to evaluate fetal cardiology programs across the globe, providing pilot data on practice components, productivity, and resource allocation. Similar to other pediatric cardiac societies, such as SCMR [1] and ASE, [2–5], that have periodically surveyed their specialties to gather benchmarking data, these survey results will help fetal cardiac programs review and evaluate their practice, identify areas for improvement, or detect programmatic needs.

The survey was distributed to fetal cardiology programs worldwide; however, 91% of respondents were from North America, and 88% possessed an academic or university affiliation. Therefore, the survey provides valuable insight into fetal cardiology practice trends from North American academically affiliated programs. Still, this sampling bias may limit the generalizability of the findings to non-academic or international programs.

Delivery resources vary across programs, directly impacting institutional delivery protocols for various CHD lesions. For programs offering childbirth services in the same hospital as pediatric cardiology, 25% of them restricted deliveries to planned cesarean sections for fetuses with complex CHD, which may increase rates of cesarean sections for fetal indications. On the other hand, one-third of the programs, mainly freestanding children's hospitals, did not have delivery services within the same hospital, requiring newborns with CHD to be transferred to the pediatric cardiology centers in that setting. Given this variability in delivery service resources, fetal cardiology programs should consider CHD risk stratification for newborn and maternal safety when developing their institution-specific delivery protocols for complex CHD lesions. Studies have shown that risk stratification algorithms based on LOC perform well in fetuses and neonates with critical congenital heart disease and can help improve patient outcomes [6–10]. While many programs (71%) reported having some delivery planning algorithms, only 51% of programs used perinatal LOC guidelines [11] to aid their perinatal management, and 13% stated they were unfamiliar with LOC guidelines. This highlights a potential area for improvement in our field.

Staffing of fetal cardiologists, fetal cardiac sonographers, and fetal cardiac care coordinators also varied amongst programs. The median number of fetal cardiologists per program was 5; however, it ranged from 1 to 22 fetal cardiologists. Similarly, the median number of fetal cardiac sonographers per program was 5, ranging from 0 to 22. The median number of fetal echocardiographic studies performed annually per program was 1,000 (IQR 580–2100) and divided amongst multiple sites capable of performing fetal echocardiographic studies (1–16 sites). Our survey, focused on programmatic assessment, did not collect data specific to individual fetal cardiologist productivity. However, based on the total annual fetal echo volume per program divided by the number of fetal cardiologists at that specific program, we roughly estimate about 220 fetal echo studies interpreted per fetal cardiologist, with the caveat that some fetal cardiologists may perform more fetal echo interpretation as compared to their colleagues in the same program. These data can help programs ensure they are properly staffed and can be used to advocate for appropriate coverage based on an individual program’s volume.

Fetal cardiac coordinators are integral members of the fetal cardiology team with a wide range of roles, including, but not limited to, care coordination, scheduling, patient counseling, delivery planning, and support of QI programs, as well as research and fetal database management. Seventy percent of programs have one or more fetal cardiac coordinators. Programs were more likely to have a QI program if they also had a fetal cardiac coordinator.

Ultrasound machines are often shared among various sites and subspecialties, with programs having a median of one machine reserved for fetal echocardiography and multiple other machines shared with pediatric cardiology or MFM/OB teams. This data can be used to help advocate for the availability of proper equipment to support a program’s volume.

We found that the use of special fetal echocardiography techniques such as early gestational fetal echocardiography (< 16 weeks gestation) [12] and acute MHO testing [13] is increasing, and programs that do not offer such services are interested in starting them. For this reason, more recently, a consensus statement was published to help programs establish and streamline MHO testing services [14]. As fetal echocardiography guidelines incorporate these newer techniques with published protocols, we expect early fetal echocardiography, and MHO will become the standard of care, though billing for such services may lag.

Fetal CHD care involves multiple disciplines, including neonatology, MFM, genetics, cardiac surgery, palliative care, social work, electrophysiology, single ventricle care, and cardiac transplantation (Fig. 3). While all these disciplines were present in more than half of the programs, unfortunately, disciplines to address parental mental health were significantly lacking, with only 38% of programs offering access to mental health counselors/psychologists and only 16% offering access to psychiatry. The association between poor parental mental health and suboptimal fetal and neonatal outcomes is well-documented [15–18]. The deficiency in maternal mental health support demonstrated by our survey highlights how fetal cardiology programs must continue to advocate for access to mental health support for our families.

Fetal databases and involvement in QI initiatives are becoming more commonplace, especially in larger volume programs and those programs supported by fetal coordinators. To guide such fetal cardiology quality improvement processes, the ACPC-QNet has published four metrics: image quality, comprehensiveness, diagnostic accuracy, and prenatal detection of severe CHD. However, participation in such national-level QI initiatives still remains low, with resource limitations reported as a significant barrier to participation. Further research efforts to establish the benefit and utility of QI programs in fetal cardiology may aid in providing the impetus for medical centers to invest resources in this important activity.

### Limitations

This study has several inherent limitations of a survey-based assessment, including sampling bias, response and non-response bias, and order bias. No public list exists of every fetal cardiology program across the world; furthermore, program structure, support, and resources vary widely in different geographic and geopolitical settings. The survey respondents came primarily from North American, academic-affiliated programs, which could suggest non-response bias. The programs that responded to the survey may be significantly different than the programs that did not respond to the survey. Therefore, the results of this study may not be applicable to all fetal cardiology programs.

A notable limitation in our design was the inability to assess workforce and productivity on an individual sonographer and physician basis; we attempted to overcome this by reporting medians. However, these should be interpreted with caution when setting productivity goals for individual physicians or sonographers because the survey cannot completely adjust for other contributing factors for each individual, such as additional work-related responsibilities, patient complexity, and patient-related factors like the need for translators in clinical practice. Additionally, the annual fetal echo volume per center did not account for the percentage of studies with abnormal findings, which may require longer scanning and counseling time compared to routine screening fetal echocardiograms. Future benchmarking studies should aim to gather such granular data if possible.

## Conclusions

This large, multicenter, international survey of fetal cardiology programs is the first to describe fetal cardiology program trends, including volume of fetal echocardiograms, practice patterns, staffing, program structure, and resource allocation. These data can be used as a framework to help fetal cardiology programs identify and advocate for areas of deficiency. Additionally, the data obtained from this survey will serve as the foundation for ongoing fetal cardiology practice development, access to care, quality of perinatal care, and outcomes assessment.

## Data Availability

No datasets were generated or analysed during the current study.

## Abbreviations

ACPC Q-Net: Adult congenital & pediatric quality network at American college of cardiology
APRN: Advanced practice registered nurse
ASE: American society of echocardiography
CHD: Congenital heart disease
CNOC: Cardiovascular neurodevelopmental outcome collaboration
FHS: Fetal heart society
FTE: Full-time equivalent
IQR: Interquartile range
IT: Information technology
LOC: Level of care
MFM: Maternal–fetal medicine
MHO: Maternal hyperoxygenation
M-mode: Motion mode
OB: Obstetrics
PA: Physician assistant
QI: Quality improvement
RN: Registered nurse
SCMR: Society of cardiovascular magnetic resonance
